# Diffusion of brain metabolites highlights altered brain microstructure in type C hepatic encephalopathy: a 9.4 T preliminary study

**DOI:** 10.3389/fnins.2024.1344076

**Published:** 2024-03-20

**Authors:** Jessie Mosso, Guillaume Briand, Katarzyna Pierzchala, Dunja Simicic, Alejandra Sierra, Ali Abdollahzadeh, Ileana O. Jelescu, Cristina Cudalbu

**Affiliations:** ^1^CIBM Center for Biomedical Imaging, Lausanne, Switzerland; ^2^Animal Imaging and Technology, École polytechnique fédérale de Lausanne (EPFL), Lausanne, Switzerland; ^3^A.I. Virtanen Institute for Molecular Sciences, University of Eastern Finland, Kuopio, Finland; ^4^Center for Biomedical Imaging, Department of Radiology, New York University Grossman School of Medicine, New York, NY, United States; ^5^Department of Radiology, Lausanne University Hospital (CHUV), Lausanne, Switzerland; ^6^Faculty of Biology and Medicine, University of Lausanne, Lausanne, Switzerland

**Keywords:** *in vivo* magnetic resonance spectroscopy, diffusion-weighted magnetic resonance spectroscopy, brain metabolism, hepatic encephalopathy, rat brain, bile duct ligation, ultra high magnetic field

## Abstract

**Introduction:**

Type C hepatic encephalopathy (HE) is a decompensating event of chronic liver disease leading to severe motor and cognitive impairment. The progression of type C HE is associated with changes in brain metabolite concentrations measured by ^1^H magnetic resonance spectroscopy (MRS), most noticeably a strong increase in glutamine to detoxify brain ammonia. In addition, alterations of brain cellular architecture have been measured *ex vivo* by histology in a rat model of type C HE. The aim of this study was to assess the potential of diffusion-weighted MRS (dMRS) for probing these cellular shape alterations *in vivo* by monitoring the diffusion properties of the major brain metabolites.

**Methods:**

The bile duct-ligated (BDL) rat model of type C HE was used. Five animals were scanned before surgery and 6- to 7-week post-BDL surgery, with each animal being used as its own control. ^1^H-MRS was performed in the hippocampus (SPECIAL, TE = 2.8 ms) and dMRS in a voxel encompassing the entire brain (DW-STEAM, TE = 15 ms, diffusion time = 120 ms, maximum *b*-value = 25 ms/μm^2^) on a 9.4 T scanner. The *in vivo* MRS acquisitions were further validated with histological measures (immunohistochemistry, Golgi-Cox, electron microscopy).

**Results:**

The characteristic ^1^H-MRS pattern of type C HE, i.e., a gradual increase of brain glutamine and a decrease of the main organic osmolytes, was observed in the hippocampus of BDL rats. Overall increased metabolite diffusivities (apparent diffusion coefficient and intra-stick diffusivity—Callaghan’s model, significant for glutamine, myo-inositol, and taurine) and decreased kurtosis coefficients were observed in BDL rats compared to control, highlighting the presence of osmotic stress and possibly of astrocytic and neuronal alterations. These results were consistent with the microstructure depicted by histology and represented by a decline in dendritic spines density in neurons, a shortening and decreased number of astrocytic processes, and extracellular edema.

**Discussion:**

dMRS enables non-invasive and longitudinal monitoring of the diffusion behavior of brain metabolites, reflecting in the present study the globally altered brain microstructure in BDL rats, as confirmed *ex vivo* by histology. These findings give new insights into metabolic and microstructural abnormalities associated with high brain glutamine and its consequences in type C HE.

## 1 Introduction

Diffusion-weighted MRI (dMRI) has emerged as a promising “super-resolution” technique that can provide information about tissue microstructure non-invasively in the order of a micron. dMRI uses the diffusion of water molecules and their interaction with tissue cellular components to generate image contrast. This signal originates from ubiquitous water molecules present in all cell types and extracellular spaces, thus limiting its specificity to any tissue compartment, cell type, or physiological phenomenon. In contrast, brain metabolites measured by magnetic resonance spectroscopy (MRS) are predominantly intracellular, and some metabolites have preferential localization within specific brain cell types (Najac et al., 2016). Myo-inositol (Ins) and glutamine (Gln) concentrations are higher in astrocytes, and N-acetylaspartate (NAA) and glutamate (Glu) concentrations are higher in neurons (Brand et al., 1993; Urenjak et al., 1993; Harris et al., 2015). Although the representation of metabolite localization in one cell type is over-simplistic *in vivo* (Rae, 2014), it constitutes a useful assumption for MRS studies.

The combination between diffusion weighting and MRS, diffusion-weighted MRS (dMRS), enables the measurement of metabolite diffusion properties, which are expected to reflect properties of intracellular space (i.e., cell-type geometry, structure, cytosol viscosity, and molecular crowding). Different dMRS modeling approaches have been proposed to quantify cell microstructure (Ligneul et al., 2024), and, among others, alterations of astrocytic morphology were observed in a mouse model of reactive astrocytes and cuprizone-fed mice as a model of glial inflammation (Ligneul et al., 2019; Genovese et al., 2021).

Type C hepatic encephalopathy (HE) is a severe neurological condition that arises as a consequence of chronic liver disease (Monfort et al., 2009; Dharel and Bajaj, 2015; Häussinger et al., 2022). In type C HE, the high ammonium delivery to the brain, due to impaired urea cycle in the cirrhotic liver, is causing Gln accumulation and the gradual release of other metabolites (Ins, taurine (Tau), total choline (tCho)) as an osmotic response (Rackayova et al., 2016; Lanz et al., 2017; Braissant et al., 2019; Cudalbu and Taylor-Robinson, 2019; Rackayová et al., 2021; Pierzchala et al., 2023). In spite of this apparent osmoregulation, a mild increase in the apparent diffusion coefficient (ADC) of water has sometimes been observed in patients with type C HE (Kale et al., 2006). It has been associated with edema without a clear consensus on its compartmentalization (Cudalbu and Taylor-Robinson, 2019; Pierzchala et al., 2023). The overall interpretation of diffusion data is difficult and sometimes controversial, as extracting quantitative metrics that characterize the underlying tissue microstructure requires modeling of the diffusion signal (Jelescu and Budde, 2017; Jelescu et al., 2020), which has not yet been proposed in type C HE. Furthermore, the presence of brain edema and/or increased brain water content is still controversial in type C HE, as type C HE, in contrast to acute HE, is characterized by lower blood ammonium values and a longer disease time course, allowing for the presence of compensatory mechanisms (Pierzchala et al., 2023). Gln synthesis in the central nervous system is largely confined to astrocytes (the site of glutamine synthetase activity) (Martinez-Hernandez et al., 1977). Thus, it has been postulated that HE is the clinical manifestation of astrocyte swelling and/or astrocyte reactivity due to increased osmotic pressure triggered by Gln accumulation, with Gln acting as an osmolyte driving water into the cells. Although the pathological role of astrocytes in animal models and humans with severe hyperammonemia and liver failure has been confirmed, it has also become clear that additional cell types in the brain are also involved in the pathogenesis of HE. To date, the direct effects of Gln accumulation on astrocytes and potentially on other cell morphology concomitant with the appearance of brain edema in type C HE are not clear, mainly due to a limited number of *in vivo* studies. An increase in brain Gln will eventually lead to cellular microstructural changes despite osmoregulation (i.e., release of other brain osmolytes). Diffusion-weighted MRS is a powerful tool to study these alterations non-invasively and *in vivo* in an animal model of type C HE.

The aim of our study was to follow *in vivo* the longitudinal evolution of brain Gln and other metabolite diffusion properties in a rat model of type C HE using dMRS, thus providing information on potential microstructural alterations during type C HE. Furthermore, histological assessment of the brain tissue was performed to validate the *in vivo* dMRS findings, and short TE ^1^H-MRS measurements in the hippocampus were performed to validate the well-known metabolic changes in type C HE.

## 2 Materials and methods

### 2.1 Animal model of chronic liver disease induced type C HE

All experiments were approved by the Committee on Animal Experimentation for the Canton de Vaud, Switzerland (VD3022.1). Adult male Wistar rats underwent bile duct ligation (BDL; Charles River Laboratories, L’Arbresle, France, 175–200 g at surgery) to create a model of chronic liver disease-induced type C HE, as previously described (Braissant et al., 2019; DeMorrow et al., 2021), and SHAM surgery as controls for histology assessment only. BDL animals were scanned longitudinally under isoflurane anesthesia (~1.5%, in a mixture of 50% oxygen and 50% air). A first scan was performed before surgery (n = 5, “week 0”), and a second scan between 6- and 7-week post-BDL (same animals, n = 5, “week 6”); the long duration of the MRI/MRS experiments did not allow us to scan all animals on the same day. For the MRI/MRS scans, all animals were placed in an in-house-built holder, with the head fixed in a stereotaxic system using a bite bar and a pair of ear bars. A small-animal monitor system (SA Instruments, New York, NY, USA) was used to monitor the body temperature (maintained at 37.7 ± 0.2°C by warm circulating water and measured with a rectal thermosensor) and the respiration rate. Blood samples of bilirubin (Reflotron Plus system, F. Hoffmann-La Roche Ltd.) and ammonium (PocketChem™ BA PA-4140) were performed before the MRI/MRS scans (before BDL and at week 6–7 post-BDL) to validate the model of chronic liver disease.

### 2.2 MRI, ^1^H-MRS and dMRS

All experiments were performed on a 9.4 T, actively shielded MRI system with a 31-cm horizontal bore (Magnex Scientific, Oxford, United Kingdom), featuring a 12-cm gradient coil insert (400 mT/m, 120 μs) interfaced to an Agilent/Varian Direct Drive console (Palo Alto, CA, USA). An in-house-built ^1^H quadrature transceiver was used (25-mm inner diameter).

**Figure 1:**
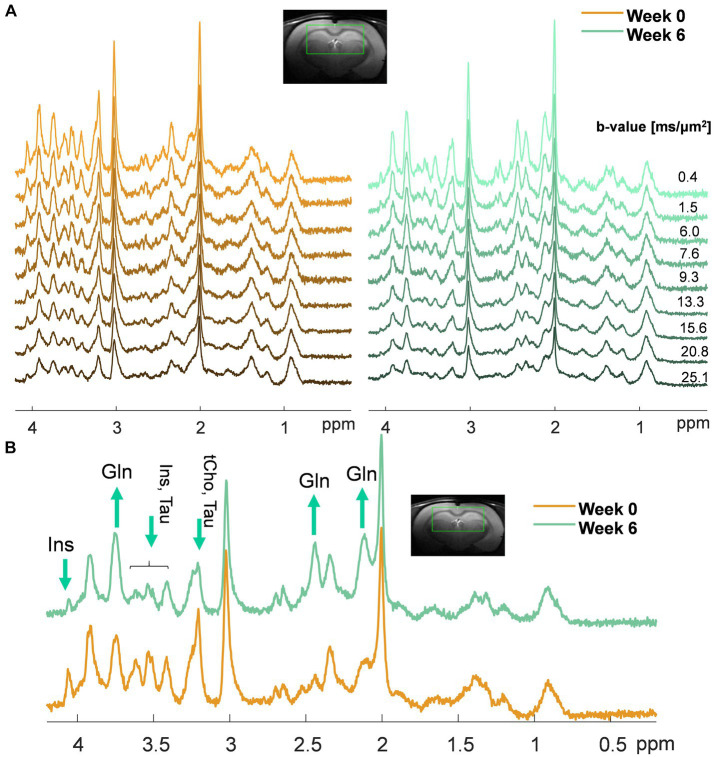
**(A)** Representative sets of diffusion-weighted spectra in one animal at week 0 (orange, left) and week 6 post-BDL surgery (green, right) acquired with diffusion-weighted STEAM. The voxel position and the *b*-values used are displayed at the top. Of note, the differences in noise level among the spectra are due to a different number of shots for each on the *b*-value (less noise at high *b*-values due to more shots); **(B)** Example of spectra acquired in the same animal at week 0 (orange) and week 6 post-BDL surgery (green) at *b* = 0.4 ms/μm^2^, highlighting with arrows the increase in brain Gln and decrease of the main brain osmolytes (i.e., Ins, tCho, Tau) observed qualitatively.

Fast T_2_-weighted images (multislice turbo-spin-echo sequence, TR = 4,000 ms, TE_eff_ = 52 ms, echo train length = 8, field of view (FOV) = 23 × 23 mm^2^, slice thickness = 1 mm, 15 slices, matrix size = 256 × 256, two averages) were acquired in the axial direction to position the volumes of interest (VOIs) for ^1^H-MRS. First, a ^1^H-MRS scan was performed in the hippocampus (2 × 2.8 × 2 mm^3^, 11.8 μL) using the SPECIAL sequence (TE = 2.8 ms, TR = 4 s, 160 shots) to measure neurometabolism, as previously described (Braissant et al., 2019). Then, dMRS data were acquired using a localized STEAM-based spectroscopic pulse sequence (Kunz et al., 2010; Ligneul et al., 2024) (TE/TM = 15/112 ms, 5 kHz spectral width, 4,096 spectral points, single shot acquisitions) in a voxel ranging from 162 to 245 μL depending on the animal. The dMRS voxel size was increased compared to the hippocampus MRS voxel due to the lower signal-to-noise ratio (SNR) in the diffusion experiments as compared to a simple MRS acquisition. FASTESTMAP (Gruetter, 1993; Gruetter and Tkác, 2000) was used for shimming, leading to water linewidths of 9–10 Hz in the hippocampus and of 18–20 Hz for the dMRS VOI (Figure 1A). Outer volume suppression blocks were interleaved with the VAPOR water suppression module. Diffusion gradients were applied simultaneously along three orthogonal directions (gradient duration δ = 6 ms, diffusion time Δ = 120 ms, direction [1,1,1]). A total of nine *b*-values (in ms/μm^2^, corrected for cross-terms (Kunz et al., 2010; Mosso et al., 2024)) with the following number of shots were acquired: 0.4 (160), 1.5 (160), 6.0 (160), 7.6 (160), 9.3 (160), 13.3 (320), 15.6 (480), 20.8 (480), and 25.1 (480).

Spectra were collected as single shots (consecutive ISIS acquisitions from SPECIAL were directly combined, with each combination being labeled as “shot” in this article) and corrected for eddy current distortions and phase and frequency drifts. Outlier shots with manifest signal drops (>50%) were removed, and all shots were averaged. Metabolite signals were quantified using LCModel (Version 6.3-1 N) combined with an *in vitro* measured metabolite basis set for ^1^H-MRS hippocampus spectra and a simulated metabolite basis set for dMRS spectra using published values of *J*-coupling constants and chemical shifts (Govindaraju et al., 2000; Govind et al., 2015). The following metabolites were included in the basis sets: alanine (Ala), ascorbate (Asc), aspartate (Asp), β-hydroxybutyrate (bHB), glycerophosphocholine (GPC), phosphocholine (PCho), creatine (Cr), phosphocreatine (PCr), glycine (Gly), GABA, glucose (Glc), Gln, glutamate (Glu), glutathione (GSH), Ins, lactate (Lac), N-acetylaspartate (NAA), N-acetylaspartylglutamate (NAAG), phosphoethanolamine (PE), scyllo-inositol (Scyllo), and Tau. In addition, an *in vivo* macromolecule spectrum acquired under the same conditions as *in vivo* ^1^H-MRS and dMRS data was included in each of the corresponding metabolite basis sets (single inversion recovery, TI = 750 ms, TR = 2,500 ms, no diffusion weighting, and metabolite residuals eliminated as described previously (Cudalbu et al., 2021; Simicic et al., 2021)). For ^1^H-MRS, metabolites with relative Cramer Rao Lower Bounds (CRLB) below 25% at week 0 for all animals were reported (selecting reported metabolites but not individual values), a purposely loose criterion to avoid filtering out low-concentrated metabolites (Kreis, 2016). For dMRS, to limit error propagation, only metabolites with relative CRLBs below 6% at the lowest *b*-value for all animals were reported.

Metabolite signal decays as a function of *b*-value were then fitted using two different approaches, using a non-linear least squares algorithm in MATLAB (*fit* function, *Trust-Region* method): First, Callaghan’s model of randomly oriented sticks (Callaghan et al., 1979) (mimicking neurites or processes) with metabolite diffusivity D_intra_ along the neurite/process:

(1) $$\frac{S}{S_{0}}=\sqrt{\frac{\pi }{4{bD}_{\mathrm{intra}}}}erf\left(\sqrt{{bD}_{\mathrm{intra}}}\right)$$

Second, the cumulant expansion at second order:

(2) $$\ln\left(\frac{S}{S_{0}}\right)=-bD+\frac{1}{6}{\left(bD\right)}^{2}K$$

Yielding the apparent diffusion coefficient D and kurtosis K, where *b* refers to the *b*-value and *erf* refers to the error function. Assuming an underlying isotropic distribution of sticks (Callaghan’s model) (Equation 1), the radius of convergence of the cumulant expansion is given by the first zero of the error function in the complex plane (Kiselev and Il'yasov, 2007), whereby assuming a diffusivity of about 0.3 μm^2^/ms, b_c_ = 19 ms/μm^2^. *b*-values up to b_c_ were used for this fit. For each metabolite, the fits were performed on the individual animal diffusion signal decay, and the fitted parameters were reported as mean and SD across animals. The fits were also performed on the group-averaged diffusion signal decay for each metabolite, yielding a mean coefficient.

A detailed table of the acquisition and processing parameters following the experts’ consensus recommendations on minimum reporting standards in *in vivo* MRS (Lin et al., 2021) is presented in Supplementary Table S1.

### 2.3 Histology assessments

#### 2.3.1 Fluorescence and Brightfield microscopy

Animals (SHAM and BDL animals) were sacrificed for histological evaluation between week 6 and 7 post-BDL. The deeply anesthetized (4% isoflurane for 5 min) animals were injected with analgesic (Temgesic [Essex Pharma], 0.1 mg/kg) before transcardiac perfusion with PBS. Due to the complexity of the experiments, additional groups of animals (in addition to the ones scanned by dMRS) were used for histological evaluation and electron microscopy, and SHAM animals were not used for electron microscopy. All animals were controlled for blood bilirubin and ammonium values to ensure that the presence and evolution of CLD were in fact reflective of the dMRS group.

#### 2.3.2 Immunohistochemistry (IHC)

Brains (SHAM n = 3, BDL n = 3) were fixed in a 4% formaldehyde PBS solution overnight at 4°C, washed with PBS, and cryopreserved in a 30% sucrose PBS solution at 4°C for 48 h. They were then embedded in Tissue-Tek^®^ O.C.T. compound and cut into 16-μm sagittal sections. Astrocyte morphological alterations were depicted using mouse monoclonal anti-GFAP antibody (MAB360 Merck Millipore) (2 h at RT, 1:100 dilution) with secondary Alexa Fluor^®^ 594-AffiniPure+ Rat Anti-Mouse IgG (H + L) antibody (415–585-166 Jackson ImmunoResearch Europe Ltd.) (1 h at RT, 1:200 dilution). Nuclei were stained with DAPI (D1306, Thermo Fisher Scientific). The stained sections were mounted with ProLong™ Diamond Antifade Mountant and covered with coverslip. Morphometric measurements (processes number/cell and processes length) were performed using Sholl analysis, as previously described (Braissant et al., 2019). A total of 200 astrocytes from each group were randomly selected and traced for all processes identified through GFAP staining. An average of 350 astrocytic processes were measured per sample, amounting to approximately 1,000 processes per group (seven slides/rat).

#### 2.3.3 Golgi-cox staining

Golgi-Cox staining was performed to unveil the detailed morphology of the CA1 hippocampus neurons (Zaqout and Kaindl, 2016). Extracted brains were directly immersed in the Golgi-Cox staining solution, then stored in the dark at room temperature for 25 days prior to being washed with PBS, and cryopreserved for 48 h in 30% sucrose in PBS at 4°C. Brains were sliced into 115-μm-thick sagittal sections using a Leica VT1200 S vibratome (25 slides/hemisphere, SHAM n = 3, BDL n = 7). After the staining procedure and dehydration, the slides were mounted with Neo-Mount (EMD Millipore). For quantitative analysis, only uniformly stained tissue with clearly apparent dendritic segments and spines was used. The surface of the neuronal soma was measured, and dendritic spines were manually counted (CA1 neurons: BDL soma ~200 cells, apical and basal dendrites ~100 each; SHAM soma ~120 cells, apical and basal dendrites ~60 each). The images were acquired using a Meiji Techno TC5600 Microscope (INFINITYX-32 camera, picture size: 6,464× 4,864 pixels). The image processing and the quantitative immunohistochemical analysis were performed with INFINITY ANALYZE 7 software (Lumenera, Canada).

#### 2.3.4 Electron microscopy (EM)

Deeply anesthetized BDL rats (n = 3) received an intraperitoneal injection of sodium pentobarbital. Afterward, a cardiac perfusion with 20 mL of isotonic PBS followed immediately with 300 mL of 2.5% glutaraldehyde and 2% formaldehyde in phosphate buffer (0.1 M, pH 7.4) was performed. The brains were removed 2 h after perfusion, and 100-μm-thick coronal sections were cut through the somatosensory cortex, striatum, hippocampus, and cerebellum (vibratome Leica VT1200; Leica Microsystems). Following a cacodylate buffer wash (0.1 m, pH 7.4), the sections were postfixed for 1 h in 1.5% potassium ferrocyanide and 2% osmium tetroxide in 0.13 M ice-cold cacodylate buffer, followed by 30 min in 2% osmium tetroxide alone, each in the same buffer, and then overnight (O/N) at 4°C in 1% uranyl acetate in water. After dehydrating in alcohol, the sections were infiltrated O/N with Durcupan resin (Fluka, Buchs, Switzerland). The sections were flat embedded between glass slides in fresh resin and left O/N at 65°C for the resin to harden. The images were acquired using a Carl ZEISS Merlin With 3View (Gatan) Scanning Electron Microscope (SEM) (current: 300 pA, voltage: 1.6 kV, image size: 6 nm/pixel, and *z*-axis: 50 nm) and analyzed with ImageJ FiJi16.

##### 2.3.4.1 Edema reconstruction

We imaged 21 × 21 × 14.75 μm^3^ of the hippocampus using the SEM method with a voxel size of 6 × 6 × 50 nm^3^, 3,500 × 3,500 × 295 voxel^3^. We applied the DeepACSON pipeline (Abdollahzadeh et al., 2021a,b) for the semantic segmentation of the extracellular edema in the acquired 3D-EM dataset. We used a small training set—six 2D planar images manually segmented for edema. The images were tiled into 350 × 350 voxel^2^ non-overlapping patches to initiate the training. To deal with the small training sets, we enhanced the performance of the networks by sequentially giving feedback as manual corrections to the network predictions. The training procedure for the networks follows the description in Abdollahzadeh et al. (2021a,b). For the instance segmentation of the extracellular edema, we applied a bottom-up percentile-based region agglomeration technique to merge over-segmented watersheds to perform instance segmentation of the intra-axonal spaces (Behanova et al., 2022).

### 2.4 Statistics

Data are presented as mean ± SD. For MRS, differences in metabolite concentration estimates were assessed with a repeated measure two-way analysis of variance (ANOVA) (Prism 5.03, GraphPad, La Jolla, CA, United States), with metabolites and disease (weeks 0 and 6) factors. For dMRS, differences in diffusion parameters based on individual animal fitting were assessed with a repeated measures two-way ANOVA on each parameter individually (ADC, AKC, D_intra_), with metabolites and disease (weeks 0 and 6) factors. For both MRS and dMRS, Bonferroni’s multi-comparisons *post-hoc* test was applied, where the number of comparisons was set to the number of metabolites passing the CRLB criteria (n = 17 comparisons for MRS and n = 7 for dMRS). For the histological measures, a two-way ANOVA with *post-hoc* Tukey HSD was used to test for statistical significance. All tests were two-tailed. The significance level in all tests was attributed as follows: ^*^p < 0.05, ^**^p < 0.01, ^***^p < 0.001, ^****^p < 0.0001.

## 3 Results

The characteristic ^1^H-MRS pattern of type C HE, i.e., a gradual increase of Gln as a result of ammonia detoxification and a decrease in the main organic osmolytes as an osmoregulatory response (Rackayova et al., 2016; Flatt et al., 2017; Braissant et al., 2019; Rackayová et al., 2021; Mosso et al., 2022), was present in the rats investigated in the current study. ^1^H-MRS in the hippocampus showed a significant increase of brain Gln (+178 ± 95%****), a decrease of Ins (−29 ± 14%**), trends of decrease for Tau, tCho, Glu, Asc, tCr (Cr + PCr), and GSH, and no difference for GABA, Lac, PE, tNAA (NAA + NAAG), and the macromolecules (Supplementary Figure S1). Alanine, aspartate, bHB, glycine, glucose, and scyllo-inositol, often poorly quantified even at high fields, were not reported as they did not survive the ^1^H-MRS CRLB criterion.

Similar patterns were observed in the dMRS voxel on low *b*-value spectra (qualitative results in Figure 1B): an increase in Gln (2.1 ppm and 3.7 ppm, observed from its relative amplitude compared to NAA), together with a decrease in the main osmolyte Ins (3.5 ppm).

Furthermore, all BDL rats displayed an increase in plasma bilirubin (from undetectable to 9.1 ± 2.1 mg/dL) and blood ammonium (from 25.9 ± 7.5 to 59.3 ± 30.9 μM) at 6-week post-BDL, both validating the BDL surgery and thus the chronic liver disease.

The quality of the acquired dMRS spectra both at week 0 and week 6 (Figure 1A) allowed the estimation of diffusion parameters of Gln, Glu, NAA, Ins, Tau, tCho (GPC + PCho), and tCr (Cr + PCr) and a fair comparison between the two time points. Supplementary Figure S2 displays the quality of the LCModel fits in a representative set of diffusion-weighted spectra acquired in one animal at weeks 0 and 6 post-BDL.

**Figure 2:**
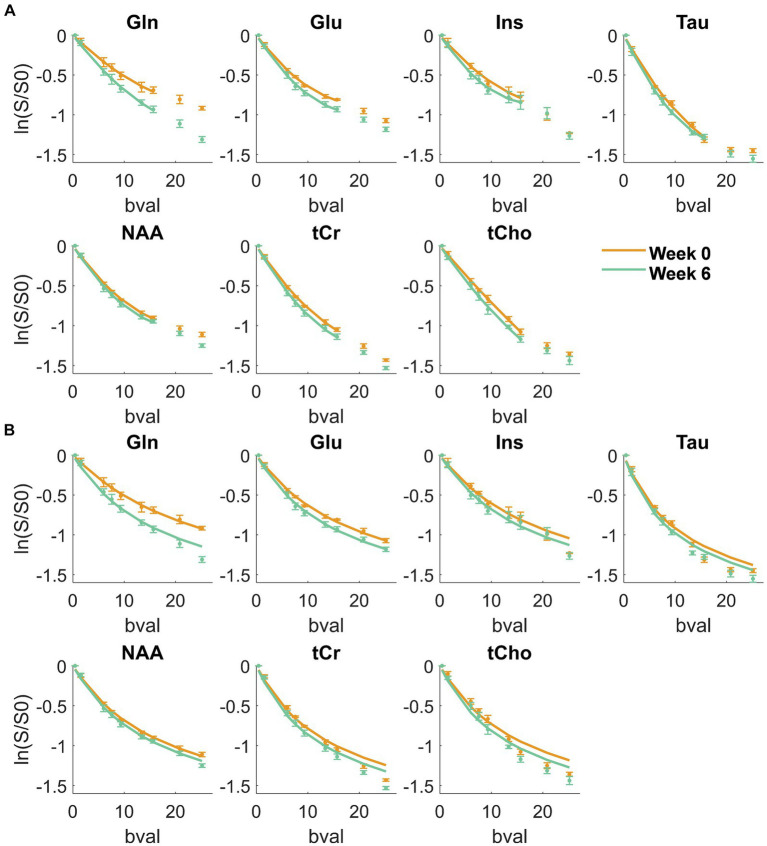
Metabolite signal diffusion decay with *b*-value, averaged over the cohort of animals at week 0 (green) and week 6 (orange) after normalization to *b* = 0.4 ms/μm^2^. Solid line: kurtosis fit up to *b* = 15.7 ms/μm^2^ **(A)** and D_intra_ fit from the randomly oriented sticks model (Equation 1) up to *b* = 25 ms/μm^2^ **(B)** Bval are in ms/μm^2^.

**Figure 3:**
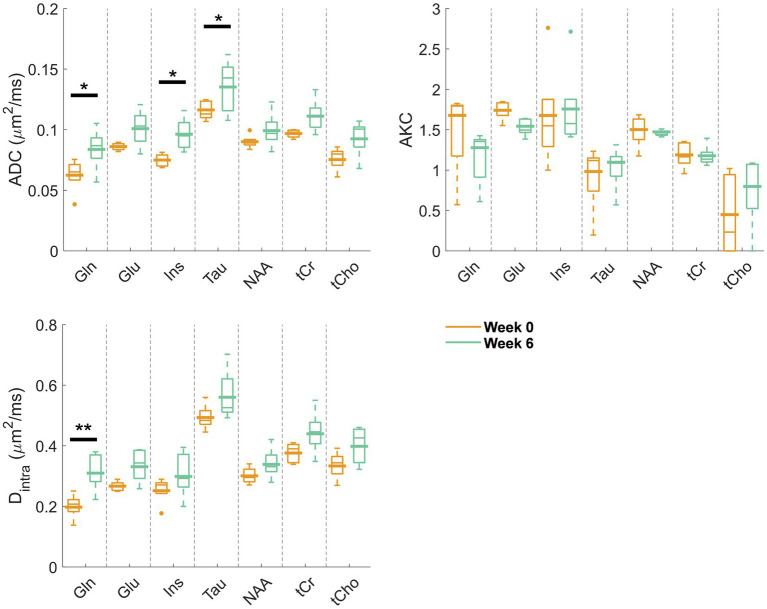
Estimated diffusion parameters from the kurtosis fit (ADC and AKC) and from the randomly oriented sticks model (Equation 1) (D_intra_) for the reliably estimated metabolites (Gln, Glu, Ins, Tau, NAA, tCr, and tCho) at week 0 (orange) and week 6 (green). Box plots: parameters fitted on the individual animal signal decays (line: median, top and bottom edges: 25th and 75th percentiles, whiskers: extreme values, dots: outliers); bold lines: parameters fitted to the mean signal decay as plotted and fitted in Figure 2. Significant differences from a two-way ANOVA (metabolite and disease factors) with a Bonferroni post-hoc test are indicated. **p < 0.01, *p < 0.05.

Figure 2 displays the metabolite signal diffusion decays with *b*-value, averaged over the cohort of animals at weeks 0 (green) and 6 (orange) after normalization to *b* = 0.4 ms/μm^2^. A good quality fit was obtained with some minor discrepancies for tCho, tCr, and Tau at high *b*-values. To further evaluate data quality, the metabolite signal diffusion decays of individual animals are also plotted in Supplementary Figures S3,S4. The derived metabolites ADC before surgery (Figure 3, first panel, orange, “week 0”) were in good agreement with results in the healthy rodent brain (Ligneul et al., 2019). After a 6-week period after surgery, an increase in intra-neurite/process diffusivity D_intra_ was measured for all metabolites (disease effect: **** with +58 ± 16% increase for Gln:**, n = 5 rats), as estimated from the sticks model (Equation 1) (Figure 3). The cumulant expansion fit (Equation 2) confirmed this trend: an increase in ADC (disease effect:****, subject-matching:* with +35 ± 14% increase for Gln:*, +29 ± 21% increase for Ins:*, +17 ± 18% increase for Tau:*, n = 5 rats) and a trend of decrease in kurtosis for some metabolites were observed, although the latter was not significant (Figure 3).

**Figure 4:**
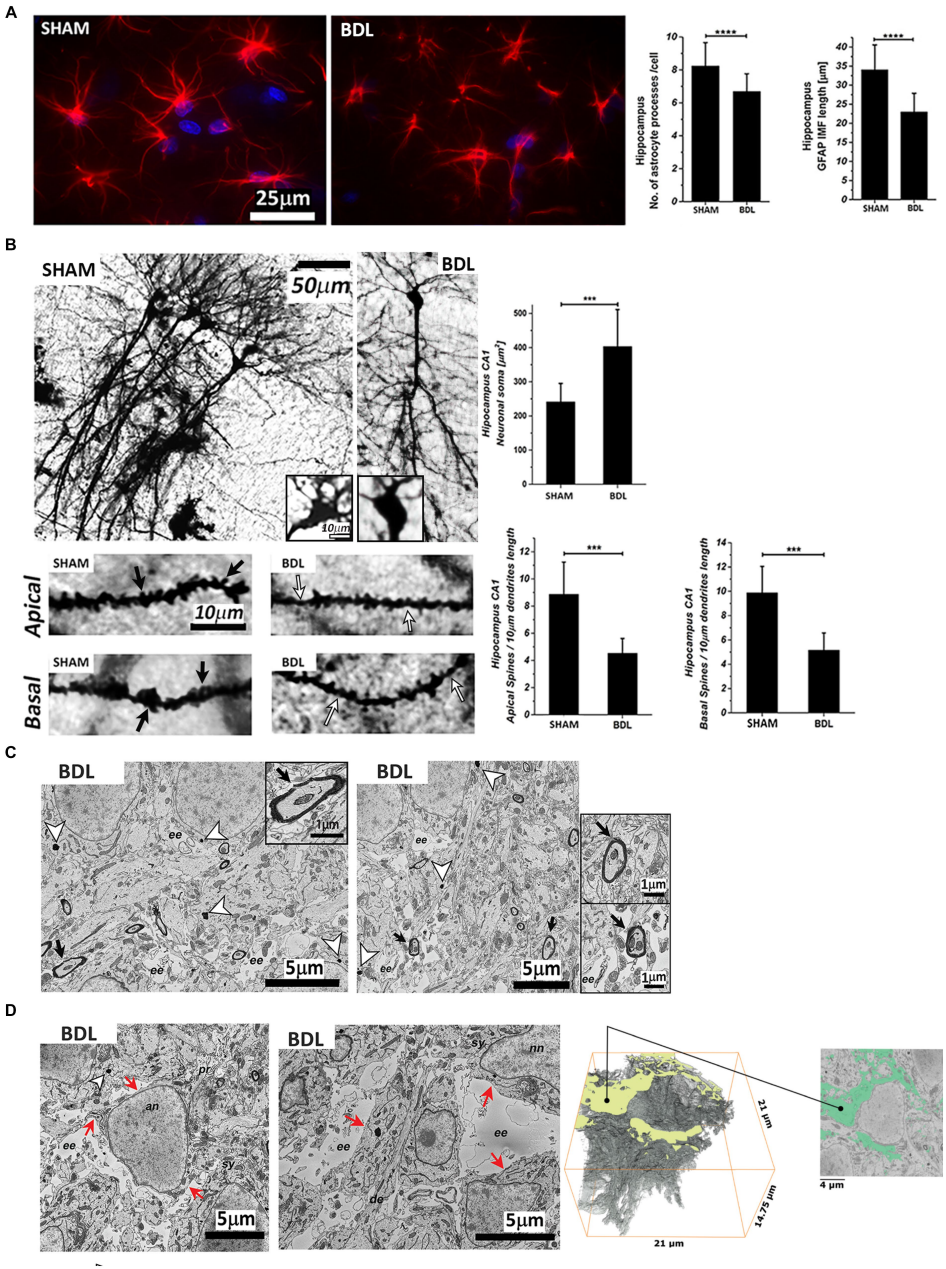
Hippocampal sections from SHAM and BDL rats. **(A)** Astrocytes stained with anti-GFAP (red) and DAPI-nuclei (blue) and morphological characterization of the number and length of processes. **(B)** Representative micrographs of Golgi-Cox staining and neuronal morphology analysis of pyramidal CA1 neurons. Black arrows indicate the spines, and the white arrows indicate spine pruning. **(C)** Analysis of the ultrastructure alterations of the hippocampus CA1 area. White arrowheads indicate granular electron-dense lipofuscin, and black arrowheads indicate myelin degeneration. **(D)** Extracellular edema reconstruction. Red arrows indicate the continuity of the plasma membrane. **(C)** and **(D)**: an—astrocyte nuclei; nn—neuronal nuclei; ee—extracellular edema; sy—synapse; se—synaptic edema; pr—processes; de—dendrite. Data are presented as mean ± SD and statistical significance (two-way ANOVA with post-hoc Tukey HSD): ^*^p < 0.05, ^**^p < 0.01, ^***^p < 0.001, ^****^p < 0.0001.

The Sholl analysis of the GFAP-stained astrocytes showed morphological alterations with a significant shortening of the processes (~30%****) and a decrease in the number of processes per cell (~18%****) at week 6 post-BDL (Figure 4A). In addition, the Golgi-Cox staining showed a significant increase in CA1 hippocampal neuronal soma surface (~65%***) and a significant loss of dendritic spines density, both apical and basal (both ~50%***) (Figure 4B). Electron microscopy of the BDL rats’ brains revealed changes in the ultrastructure, as shown in Figures 4C,D, which were not observed in the healthy rat brain (Nahirney and Tremblay, 2021). Electron-dense lipofuscin granules were found in the perikaryal cytoplasm of neurons and in the cytoplasm of astrocytes, and aggregates are indicated in Figure 4C, together with elongated/fused mitochondria. A pattern of myelin sheath degeneration/breakdown was also observed (Figure 4C). The hippocampus was characterized by loss of tissue integrity and enlarged extracellular spaces, indicating increased extracellular water content in the area surrounding the astrocytes (Figure 4D). Automatic segmentation of extracellular space allowed reconstruction of the edema volume (938.743 μm^3^), corresponding to 14.5% of the whole dataset volume (6504.75 μm^3^).

## 4 Discussion

The present study describes the first *in vivo* implementation of single-voxel MRS, dMRS, and histology in rats with chronic liver disease-induced HE. It evaluates the potential of dMRS to highlight microstructural changes in the rat brain with type C HE through the measurement of metabolite diffusion properties. ^1^H-MRS probed an increase in Gln and a decrease in the main osmolytes (Ins) in the hippocampus of BDL rats at 6-week post-surgery, indicating the presence of osmotic stress. The additional use of dMRS in the same animals enabled the characterization of changes in the diffusion behavior of brain Gln, Glu, Ins, Tau, tCr, and tCho (an overall increase as a disease effect, with Gln, Ins, and Tau showing significant changes), highlighting the presence of microstructural changes in this animal model, which were validated by different histological measures (astrocytes: decreased number and length of GFAP-stained intermediate filaments; neurons: decreased density of dendritic spines; and enlarged extracellular spaces). Taken together, our data suggest a loss of tissue integrity, providing new insights into metabolic and microstructural alterations linked to increased brain Gln and its consequences in type C HE. Furthermore, our results confirm that type C HE is characterized by complex and multicellular alterations that go beyond the hypothesis of intracellular edema, with astrocytes being the only target.

### 4.1 Brain metabolism alterations in the hippocampus

The increase in brain Gln in the hippocampus, mainly due to increased blood ammonium as a consequence of chronic liver disease, led to an osmotic imbalance resulting in a gradual decrease of other brain osmolytes (Ins), and contributed to morphological astrocytic alterations (shortening of process length together with a decrease in their number) (Häussinger et al., 2000), among other mechanisms. Of note, osmotic stress is not the sole mechanism involved in type C HE; oxidative stress and inflammation are complementary mechanisms acting synergistically (Simicic et al., 2022; Andersen and Schousboe, 2023; Pierzchala et al., 2023). Consistent with our previous findings (Braissant et al., 2019), we also identified some trends (not significant) in the changes of other metabolites. These changes included a decrease in Tau and tCho due to the osmotic response, the neurotransmitter Glu, and the antioxidants Asc and GSH. Moreover, the EM-observed intracellular accumulation of lipofuscin aggregates is a sign of lipid peroxidation and thus the presence of oxidative stress, which is in agreement with our previous studies (Pierzchala et al., 2022; Simicic et al., 2022), and we observed herein a decrease in antioxidants, a sign of redox homeostasis alterations. The same trends in metabolite changes were also observed in the bigger VOI used for dMRS. The quantification of these data was not used for characterizing the brain metabolism due to the longer TE of the dMRS sequence and the lack of T_2_ corrections for water and metabolites.

### 4.2 Brain microstructural alterations revealed by dMRS and histology

In the BDL group at week 6, increased diffusivities of brain Gln and of the main brain osmolytes (Ins, Tau) were observed when compared to week 0. This observation is a possible consequence of the osmotic stress caused by intra-astrocytic Gln increase: brain osmolytes may be temporarily present in the extracellular space, therein experiencing freer diffusion compared to intracellular space, before being cleared out, as supported by the steady-state net decrease of osmolyte concentrations observed with MRS. The electron microscopy results revealed a loss of tissue integrity and enlarged extracellular spaces in the hippocampus, indicating increased extracellular water content in the area surrounding the astrocytes.

Furthermore, the increased diffusivities of Gln and Ins in the BDL rats, metabolites assumed to be glial markers (Martinez-Hernandez et al., 1977; Häussinger et al., 1994), may also reflect astrocyte alterations following the strong Gln increase. Changes in diffusivity *in vivo* are usually associated with microstructural changes (Najac et al., 2014, 2016; Palombo et al., 2018; Ligneul et al., 2019; Genovese et al., 2021), and the diffusion time used in the present study (120 ms, characteristic 2D diffusion length of $\sqrt{4D\Delta }=8.5\mathrm{\mu m}\text{,}$ assuming D=0.15 μm^2^/ms) is likely to probe metabolite diffusion along fibers (astrocytic processes or neuronal dendrites) rather than confinement in cell bodies, as shown with dMRS in the human (Najac et al., 2016) and macaque brain (Najac et al., 2014). These dMRS findings are supported by the GFAP histological observations (i.e., decreased length and number of astrocytic processes) pointing toward a less restricted and ramified cellular architecture, explaining increased diffusivities for astrocytic metabolites in the BDL rats. A recent study showed increased serum GFAP levels in cirrhotic patients (Gairing et al., 2023), suggesting that the presence of astrocyte injury and astrocyte activation are two mechanisms that may lead to increased serum GFAP concentrations. It is worth mentioning that, using dMRS in a model of reactive astrocytes, Ins has been revealed as a specific intra-astrocytic marker whose diffusion closely reflects astrocytic morphology, enabling the non-invasive detection of astrocyte hypertrophy (Ligneul et al., 2019). In addition, the diffusion of astrocytic metabolites can mirror their altered morphology and pro-inflammatory phenotype (de Marco et al., 2022), since, during neuroinflammation, both astrocytes and microglia undergo metabolic, functional, and morphological changes (Heneka et al., 2014). In our previous studies, significantly elevated levels of IL-6 and reactive oxygen species were observed in the brains of BDL rats as compared to the SHAM animals (Pierzchala et al., 2022), suggesting the presence of neuroinflammation. IL-6 levels, together with oxidative stress, have also been associated with increased blood–brain barrier permeability, allowing neurotoxins to enter the brain and impair neurological functions (Simicic et al., 2022). Recent studies promoted dMRS as a tool sensitive to glial cytomorphological changes induced by inflammation following LPS administration in humans (de Marco et al., 2022) or in cuprizone-fed mice (Genovese et al., 2021), where Ins and tCho apparent diffusion coefficients were significantly elevated. Following these studies, tCho diffusivity changes were related to the presence of inflammation, even though tCho has a limited specificity for glial cells. Similarly, an increased diffusivity for tCho (a trend of ~20% increase) was measured in this study, possibly reflecting here also the presence of neuroinflammation, as shown previously in BDL rats *ex vivo* (Pierzchala et al., 2022). Neuroinflammation will impact the astrocyte cytoskeleton, which may lead to an increase in intracellular and extracellular space, as observed in our study. Furthermore, the EM data depicted a breakdown of myelin sheaths and myelin outfolding formation in BDL rats, which could also impact tCho diffusivity as tCho is required for membrane phospholipid synthesis and myelination (Zeisel et al., 1986), although only a few studies have validated the association between myelin status and tCho (Laule et al., 2007; Rae, 2014, Skripuletz et al., 2015).

Glu and NAA, both expected to be preferentially located in neurons (MOFFETT et al., 2007; Fendt and Verstreken, 2017), exhibited a trend of increased diffusivity in BDL rats at week 6 post-BDL. Golgi-Cox measures probed an increased soma surface of CA1 hippocampal neurons and a loss of dendritic spines density (which is made of filamentous actin cytoskeleton; Hering and Sheng, 2001) in BDL rats. Numerical simulations (Palombo et al., 2018) have suggested that decreased dendritic spines density would increase the ADC of neuronal metabolites, consistent with the trend of increased Glu and NAA diffusivity observed herein. Furthermore, additional studies using two-photon microscopy on brain slices (Santamaria et al., 2006, 2011) have shown that the diffusional characteristics of dendrites are greatly affected by the dendritic spines density, being slower in the dendrite with the higher density of spines due to anomalous diffusion, and significantly faster in smooth/low spines density dendrites. Of note, Glu is the main precursor of Gln synthesis in the astrocytes, and the observed increased diffusivity trend can also reflect the reduced number and shortening of astrocyte processes. Total Cr also showed a trend of increased diffusivity in this study. Creatine is located in most cell types, has different roles in energy metabolism and cytoprotection, and also appears to act in osmoregulation and neurotransmission (Rackayova et al., 2017; Braissant et al., 2019).

Metabolite kurtosis coefficients overall tended to decrease in the BDL group, suggesting that the intracellular space might be less heterogeneous with reduced structural disorder. Although previous numerical simulations have shown that the number of processes departing from the soma has almost no influence on the measured ADC at any diffusion time (Palombo et al., 2016), the former, observed here by histology, might have an influence on D_intra_, which is higher in HE rats compared to control rats for most metabolites. Overall, we believe that increased diffusivities in type C HE rats versus control rats reflect (1) intracellular space alteration with reduced structural disorder, supported by decreased neuronal spines density, decreased length of astrocyte processes and number of ramifications shown by histology, and (2) a higher contribution of extracellular space diffusion in BDL rats compared to controls due to osmolytes leaving the cells counteracting intracellular Gln increase.

### 4.3 Limitations

dMRS is a challenging measurement, and different factors might affect the estimated diffusion metrics (Ligneul et al., 2024). In the present study, motion artifacts due to simple linear translational motion were compensated on individual shots by phase correction, while data affected by rotational and compressive motion were discarded in the outlier removal process. Consequently, we do not expect any significant effect of motion on the calculated metabolite diffusion metrics, i.e., an overall overestimation. Additionally, a change in metabolite concentration is unlikely to affect diffusivity through a change in cytosol viscosity given the small metabolite concentrations (1–10 M) compared to water (45–50 M) (Kinsey et al., 2011).

For some metabolites (i.e., Tau, tCr, and tCho), the sticks model (Equation 1) showed some discrepancies with the measured data at high *b*-values. These discrepancies may result from a poorer LCModel spectral fit quality at higher *b*-values, exemplified by the high inter-animal variability of estimated concentrations for the highest *b*-values (Supplementary Figures S3,S4). Such variability might be partially alleviated by the use of simultaneous 2D fitting of the spectral and diffusion dimensions (Adalid et al., 2017).

The strict CRLB criterion for dMRS ensures a fair comparison between the groups. The use of each animal as its own control with a scan before the BDL surgery was beneficial as it ruled out possible inter-animal differences in brain microstructure or metabolism that could have biased the group comparison. A good concordance between individual and group-average fit for diffusion estimates was obtained in the current study. The possibility of fitting diffusion coefficients on individual animal signal decays provides an error estimation better representing the group dispersion than the one evaluated from the group-averaged signal decay. dMRS is also characterized by an overall low signal-to-noise ratio (SNR) compared to a simple MRS acquisition. In the present study, the LCModel SNR ranged from 15–20 to 45 depending on the *b*-value, guiding the choice of the randomly oriented sticks model (Equation 1) instead of the randomly oriented cylinder model (i.e., fitting, in addition, the radius of processes) (Vangelderen et al., 1994). This finding is highlighted in a recent dMRS consensus article showing that fitting the cylinder model would require a higher SNR and higher *b*-values than what was accessible in the present study (Ligneul et al., 2024). Finally, the increased brain Gln combined with the relatively short TE (15 ms) and high magnetic field allowed us to report brain Gln diffusivity for the first time.

Our study reports an overall increased diffusivity for all investigated metabolites, which was confirmed by histological measures. However, additional studies with an increased number of samples would be required to confirm this trend together with EM data on SHAM animals. dMRI could provide additional information with respect to dMRS, the former also informing on the extracellular space and on exchange between intracellular and extracellular spaces. An increased membrane permeability in BDL rats would also contribute to reduced compartmentalization (intracellular vs. extracellular) of metabolites (Ins, Gln, and Tau) and faster diffusion, which could be evaluated from joint dMRS and dMRI acquisitions in future studies. Future dMRS studies in this animal model should focus on targeting a specific brain region (the dMRS voxel here included several brain regions): brain regional differences in the neurometabolic profiles of BDL rats have been suggested, with the cerebellum exhibiting a stronger Gln increase than other brain regions (Simicic et al., 2019).

## 5 Conclusion

In conclusion, this study highlights the potential of dMRS as a unique tool to non-invasively monitor neuronal and astrocytic structural alterations in the rat model of type C HE via the measurement of cell-specific metabolite diffusion properties. The increased diffusivity and reduced kurtosis in BDL versus control rats, measured *in vivo* with dMRS, are consistent with an altered microstructure probed *ex vivo* by fluorescence, brightfield, and electron microscopy. Overall, dMRS evidenced that type C HE is characterized by complex and multicellular alterations beyond astrocyte swelling and holds enormous potential for future HE studies.

## Data availability statement

The raw data supporting the conclusions of this article will be made available by the authors, without undue reservation.

## Ethics statement

The animal study was approved by Committee on Animal Experimentation for the Canton de Vaud, Switzerland (VD 3022.1). The study was conducted in accordance with the local legislation and institutional requirements.

## Author contributions

JM: Formal analysis, Methodology, Software, Visualization, Writing – review & editing. GB: Formal analysis, Methodology, Software, Visualization, Writing – review & editing. KP: Formal analysis, Methodology, Visualization, Writing – review & editing, Investigation, Writing – original draft. DS: Methodology, Writing – review & editing. AS: Methodology, Writing – review & editing, Formal analysis, Visualization. AA: Formal analysis, Methodology, Writing – review & editing, Visualization. IJ: Methodology, Software, Writing – review & editing. CC: Methodology, Software, Writing – review & editing, Conceptualization, Formal analysis, Funding acquisition, Investigation, Project administration, Resources, Supervision, Visualization, Writing – original draft.
