# Supplementary material for: Diffusion of brain metabolites highlights altered brain microstructure in type C hepatic encephalopathy: a 9.4 T preliminary study
Source: Front Neurosci. 2024 Mar 20;18:1344076. doi: 10.3389/fnins.2024.1344076 (PMC10987698; doi:10.3389/fnins.2024.1344076)
Supplement: Supplementary file 1 [file Data_Sheet_1.pdf]

Supplementary Material

Diffusion of brain metabolites highlights altered brain microstructure in type C hepatic encephalopathy: a 9.4T preliminary study

Jessie Mosso[1,2,†], Guillaume Briand[1,2,†], Katarzyna Pierzchala[1,2], Dunja Simicic[1,2], Alejandra Sierra[3], Ali Abdollahzadeh[4], Ileana Jelescu[5,6] and Cristina Cudalbu[1,2*]

[1] CIBM Center for Biomedical Imaging, Switzerland

[2] Animal Imaging and Technology, École polytechnique fédérale de Lausanne (EPFL), Lausanne, Switzerland

[3] A.I. Virtanen Institute for Molecular Sciences, University of Eastern Finland, Kuopio, Finland

[4] Center for Biomedical Imaging, Department of Radiology, New York University Grossman School of Medicine, New York, NY, USA

[5] Department of Radiology, Lausanne University Hospital (CHUV), Lausanne, Switzerland

[6] Faculty of Biology and Medicine, University of Lausanne, Lausanne, Switzerland

† These authors contributed equally to this work and share first authorship

*** Correspondence:**
Cristina Cudalbu
cristina.cudalbu@epfl.ch

1 Supplementary tables

**1. Hardware**	**9.4 T rodent data**
**a. Field strength [T]**	9.4 T
**b. Manufacturer**	Varian
**c. Model (software version if available)**	Direct Drive Console
**d. RF coils: nuclei (transmit/receive), number of channels, type, body part**	Homemade quadrature 2 loops surface coil

**e. Additional hardware**	Gradient strength: 400mT/m
**2. Acquisition**	
**a. Pulse sequence**	Diffusion-weighted STEAM
**b. Volume of Interest (VOI) locations**	Full brain
**c. Nominal VOI size [cm^3, mm^3]**	162-245 μL
**d. Repetition Time (TR), Echo Time (TE), mixing time (TM)**	TE: 15 ms<br>TM: 112 ms<br>TR: 4000 ms
**e. Total number of Excitations or acquisitions per spectrum**	160-480 shots per b-value<br>9 b-values from 0.4 to 25.2 ms/μm^2
**f. Additional sequence parameters (spectral width in Hz, number of spectral points, frequency offsets)**	5000 Hz<br>4096 points<br>Duration of diffusion gradients: 6ms<br>Diffusion time: 120ms
**g. Water Suppression Method**	VAPOR
**h. Shimming Method, reference peak, and thresholds for "acceptance of shim" chosen**	FASTMAP, target LW: 18-20 Hz
**i. Triggering or motion correction method**	None
**3. Data analysis methods and outputs**	
**a. Analysis software**	LCModel version 6.3-1N

**b. Processing steps deviating from quoted reference or product**	ECC using the water signal, B_0 drift, outlier removal based on visual inspection
**c. Output measure**<br><br>**(e.g. absolute concentration, institutional units, ratio)**	Concentrations in arbitrary units
**d. Quantification references and assumptions, fitting model assumptions**	The basis sets include an in vivo acquired MM spectrum<br><br>NUNFIL 2048<br><br>*NRATIO = 0*<br><br>NOMIT 8: lipids, Gua, Ser, -CrCh2, bHB<br><br>Fit region: 0.2-4.2ppm<br><br>DKNTMN 0.25
**4. Data Quality**	
**a. Reported variables**<br><br>**(SNR, Linewidth (with reference peaks))**	SNR: not reported as different averages per b-value but checked in the LCModel output<br><br>LW: not reported but checked in the LCModel output
**b. Data exclusion criteria**	None
**c. Quality measures of postprocessing Model fitting (e.g. CRLB, goodness of fit, SD of residual)**	SD across animals, LCModel SD%
**d. Sample Spectrum**	Figure 1

Supplementary Table S1 – Minimum reporting standards in MRS, from ref (Lin *et al.*, 2021)

2 Supplementary figures

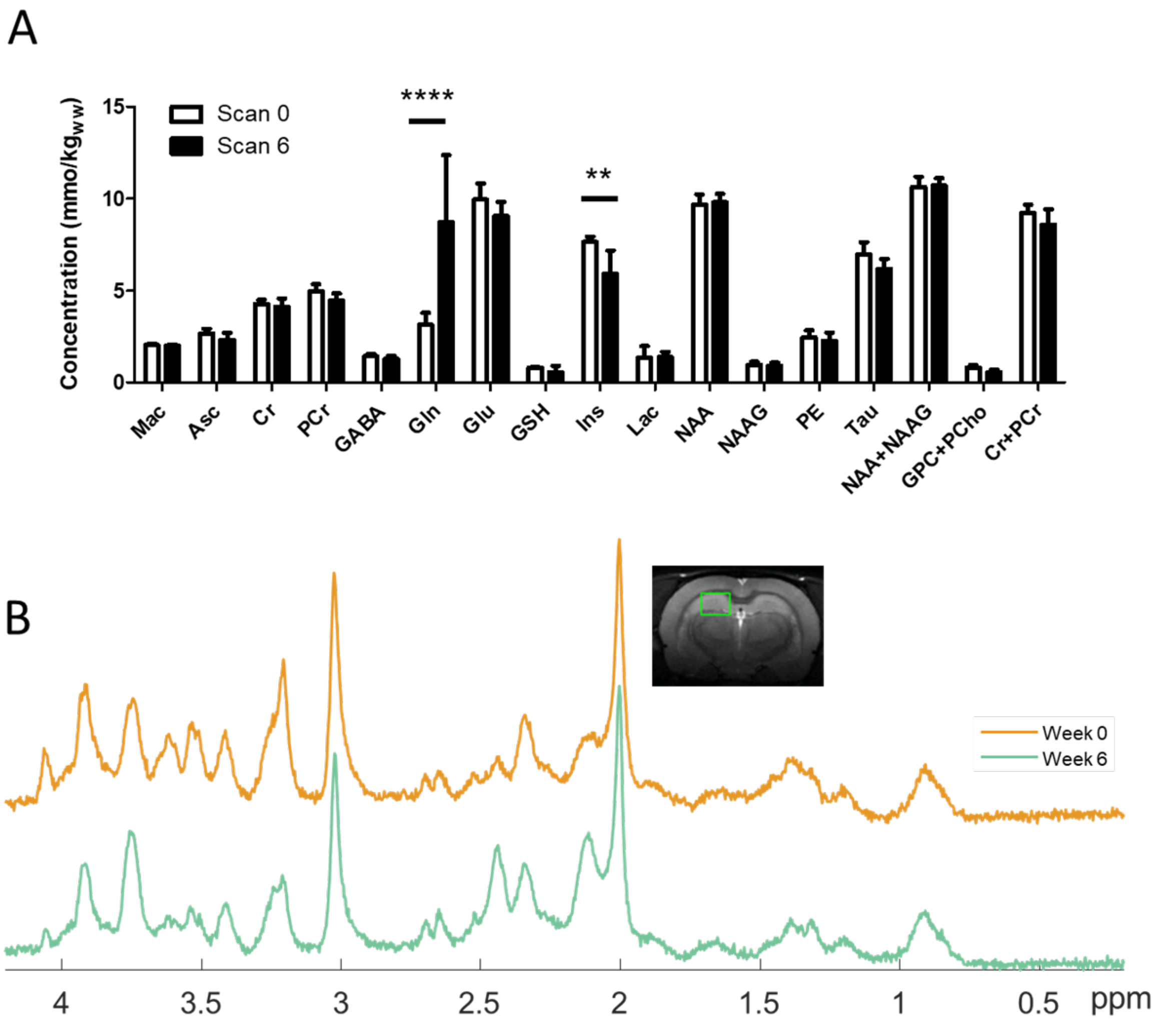

Supplementary Figure 1: (A) Metabolite concentration estimates in the hippocampus, referenced to water (mean±SD, n=5), significant differences from a two-way ANOVA (metabolite and disease factors) with Bonferroni post-hoc test. **(B)** Representative spectra in the hippocampus of one animal at weeks 0 and 6. ****: $p<0.0001$, **: $p<0.01$.

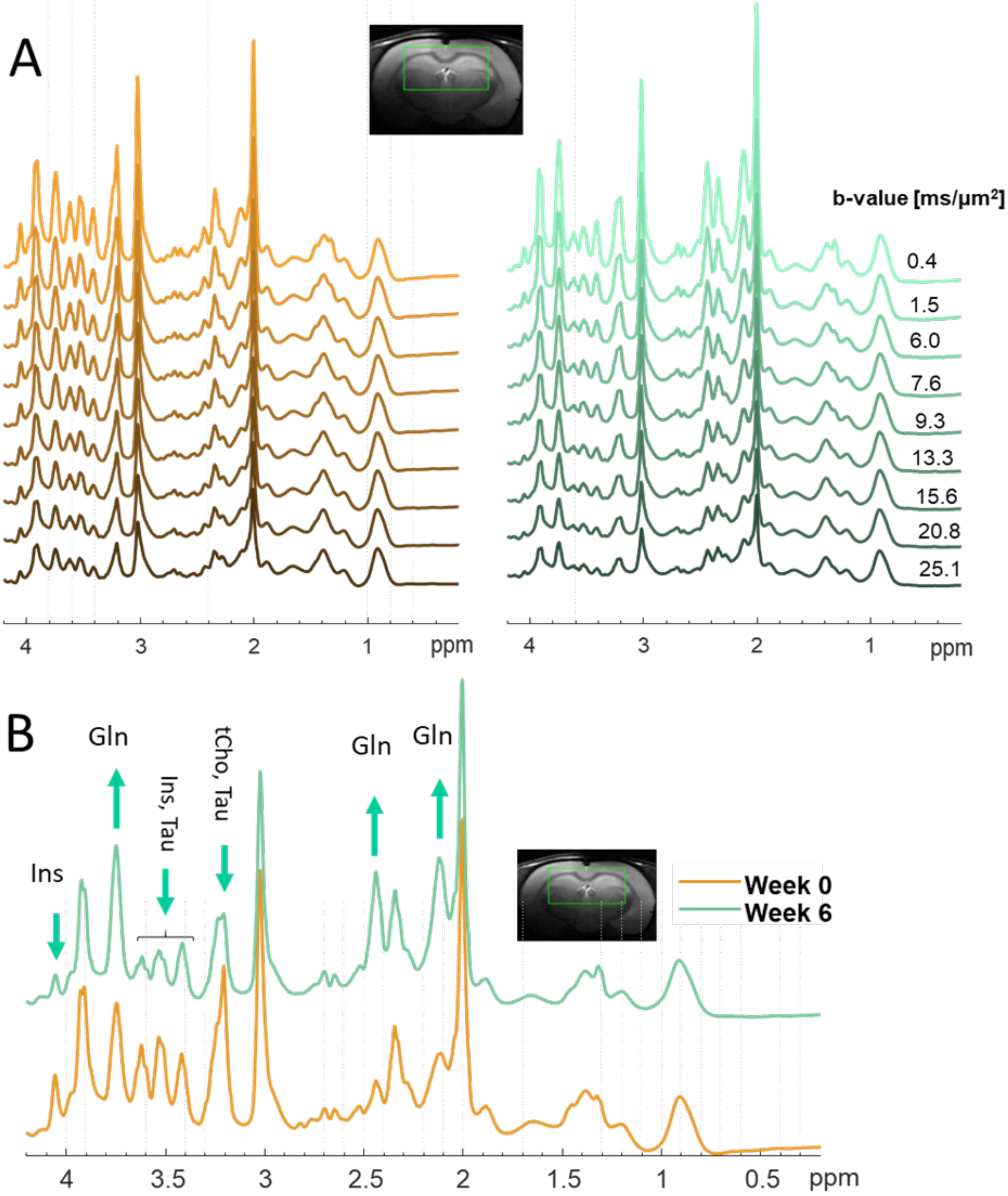

Supplementary Figure 2: **(A)** Representative sets of diffusion-weighted fitted spectra (LCModel fits) in one animal at week 0 (orange, left) and week 6 post BDL surgery (green, right) acquired with DW-STEAM. The voxel position and the b-values used are displayed at the top. **(B)** Example of fitted spectra (LCModel fits) acquired in the same animal at week 0 (orange) and week 6 post BDL surgery (green) at b = 0.4 ms/µm^2 highlighting with arrows the increase in brain Gln and decrease of the main brain osmolytes (i.e. Ins, tCho, Tau).

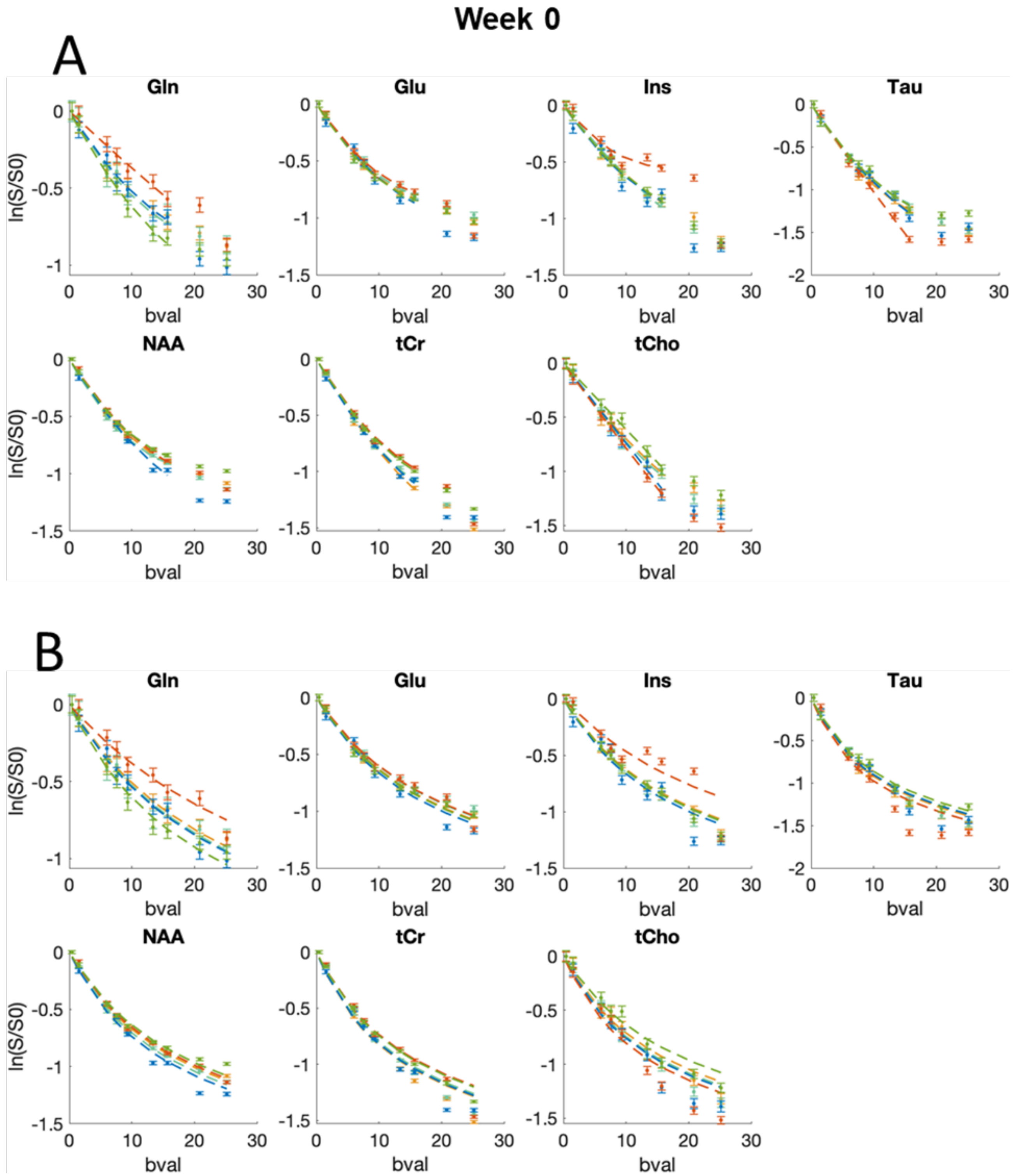

Supplementary Figure 3: Metabolite signal diffusion decay with b-value, for each individual animal at week 0 after normalization to the b = 0.4 ms/μm^2. Dashed line: kurtosis fit up to b = 15.7 ms/μm^2 **(A)** and D_{intra} fit from the randomly oriented sticks model up to b = 25 ms/μm^2 (**B**). bval in ms/μm^2.

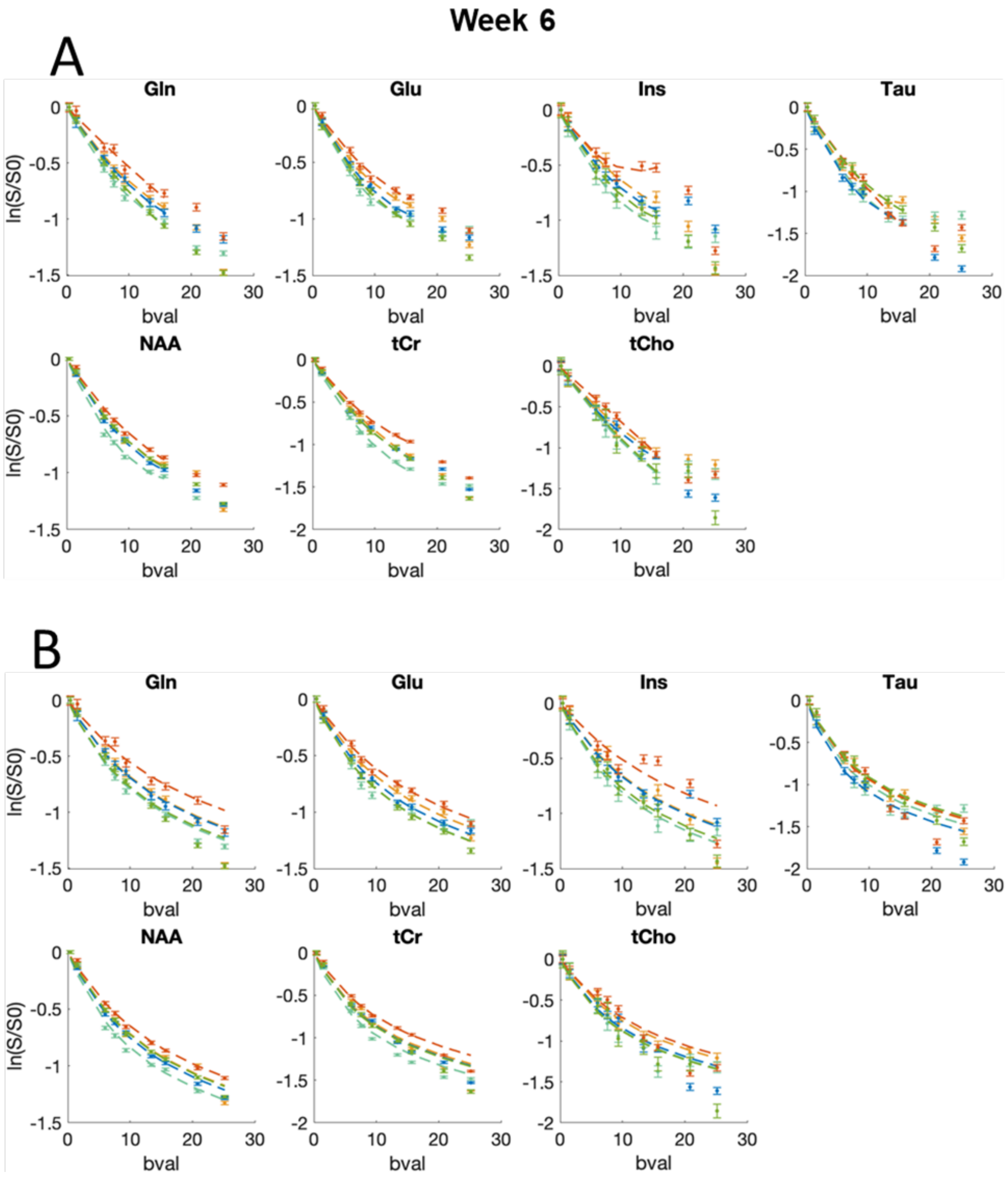

Supplementary Figure 4: Metabolite signal diffusion decay with b-value, for each individual animal at week 6 after normalization to the b = 0.4 ms/µm^2. Dashed line: kurtosis fit up to b = 15.7 ms/µm^2 **(A)** and D_{intra} fit from the randomly oriented sticks model up to b = 25 ms/µm^2 (**B**). bval in ms/µm^2.

3 References

Lin, A. *et al.* (2021) 'Minimum Reporting Standards for in vivo Magnetic Resonance Spectroscopy (MRSinMRS): Experts' consensus recommendations', *NMR in biomedicine*, 34(5), p. e4484. Available at: https://doi.org/10.1002/nbm.4484.
